# Invasive cervical resorption of central incisor during orthodontic treatment

**DOI:** 10.1590/2177-6709.25.6.049-058.oar

**Published:** 2020

**Authors:** Gabriela Meyge de Brito, Paulo Sergio Flores Campos, Ana Carolina Ramos Mariz, Diana Simões, Andre Wilson Machado

**Affiliations:** 1 Universidade Federal da Bahia, Programa de Pós-graduação em Odontologia e Saúde (Salvador/BA, Brazil).; 2 Universidade Federal da Bahia, Departamento de Radiologia Odontológica (Salvador/BA, Brazil).; 3 Universidade Federal da Bahia, Departamento de Ortodontia (Salvador/BA, Brazil).

**Keywords:** Root resorption, Maxillary expansion, Tooth movement, Ectopic tooth eruption

## Abstract

**Introduction:**

Invasive cervical resorption (ICR) is a relatively rare type of ERR (External Root Resorption), in which a localized resorption begins in the cervical area of the tooth, below the epithelial junction and above the ridge crest.

**Objective:**

Describe the clinical case of an 11-year-old boy with no dental trauma history, presenting moderate crowding and ectopic eruption of the maxillary right central incisor. He had been undergoing orthodontic treatment elsewhere, and his family was dissatisfied with the results.

**Description:**

A new treatment was indicated, which included rapid maxillary expansion followed by extraction of four premolars. During routine panoramic evaluation, a radiolucid image was detected and a periapical radiograph was requested. At this point, an ICR of the maxillary right central incisor was found. The treatment was cautiously finalized and despite the use of light forces, central incisor was severally compromised by ICR and was therefore extracted.

**Conclusion:**

This clinical example discusses the importance of routine radiographs for the early diagnoses of ICR.

## RESUMO

**Introdução:**

A reabsorção cervical invasiva (RCI) é um tipo relativamente raro de reabsorção radicular externa (RRE), no qual uma reabsorção localizada começa na região cervical do dente, abaixo da junção epitelial e acima da crista marginal.

**Objetivo:**

Descrever o caso clínico de um menino com 11 anos de idade, sem histórico de trauma dentário, apresentando apinhamento moderado e erupção ectópica do incisivo central superior. Ele realizava tratamento ortodôntico em outro local, mas sua família estava insatisfeita com os resultados.

**Descrição:**

Foi iniciado um novo tratamento, que incluiu a expansão rápida da maxila, seguida pela extração de quatro pré-molares. Durante a avaliação de rotina da radiografia panorâmica, uma imagem radiolúcida foi detectada, e uma radiografia periapical da região foi solicitada, sendo encontrada uma RCI no incisivo central superior direito. O tratamento foi finalizado com cautela e, apesar do uso de forças leves, o incisivo central estava seriamente comprometido pela RCI e, portanto, precisou ser extraído.

**Conclusão:**

Esse exemplo clínico discute a importância das radiografias de rotina para o diagnóstico precoce da RCI.

## INTRODUCTION

During orthodontic treatment, several side effects might occur. One of the most challenging problems related to tooth movement consists of external root resorption (ERR).^1^ Invasive cervical resorption (ICR) is a relatively rare type of ERR, in which a localized resorption begins in the cervical area of the tooth, below the epithelial junction and above the ridge crest.^2^ ^,^ ^3^

The damage to the tooth structure resulting from the ICR varies widely, and the tooth may become progressively weak. In severe and/or late diagnosed cases, extraction of the tooth is the remaining alternative.^3^ Since the occurrence of ICR is clinically asymptomatic, periodic radiographic examination is an important tool for its early diagnoses and treatment.^4^

Despite the unknown etiologic factors involved in the process of ICR, its occurrence has been related to intracoronal bleaching, trauma and orthodontic treatment.^5^ ^-^ ^7^ The relationship between orthodontic treatment and ERR has been widely discussed, but scientific literature still lacks elucidation about the exact mechanism by which the orthodontic forces might cause ICR.^7^ ^,^ ^8^

The aim of this article is to present a rare clinical example, describing the case of an 11-year-old male patient with a Class I, moderate crowding and ectopic eruption of the maxillary right central incisor, later diagnosed with ICR. This clinical example also discusses the possible relationship between orthodontic tooth movement and ICR, treatment options and the importance of routine radiographic exams during orthodontic therapy.

## DIAGNOSIS AND ETIOLOGY

The patient, an 11-year-old boy sought treatment at a private orthodontic clinic since his family was dissatisfied with the orthodontic treatment he was undergoing. The patient was in good general health and reported no accidental dental trauma. 

**Figure 1:**
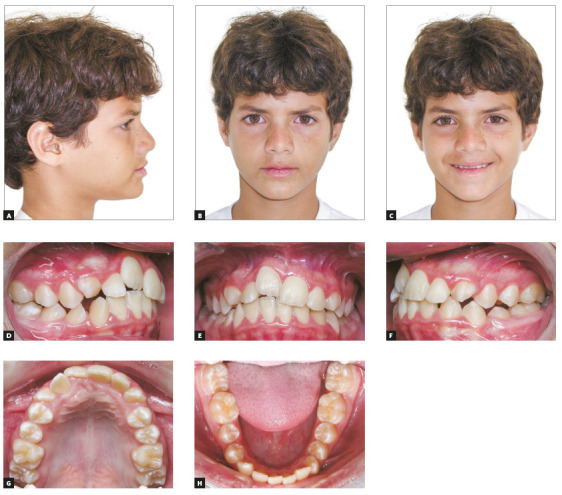
Pretreatment facial and intraoral photographs.

**Figure 2:**
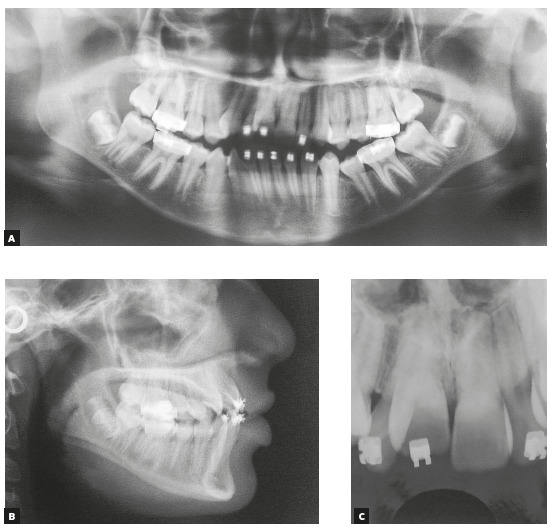
Initial panoramic, lateral, and periapical radiographs.

Intraoral photographs showed a Class I malocclusion, moderate crowding in the maxillary and mandibular arches. The maxillary right central incisor was in infraocclusion and the maxillary right lateral incisor had severe palatal root torque (Fig 1). The model discrepancy was - 4.0 mm at the mandibular arch and - 5.0 mm at maxillary arch. The panoramic radiograph showed absence of the maxillary left third molar, and the lateral radiograph (Fig 2) with cephalometric measurements indicated well positioned incisors, maxilla and mandible, and a well-balanced vertical skeletal pattern (Table 1). Maxillary incisors periapical radiograph revealed apical root rounding. The patient reported duration of 3 years and 5 months of the previous treatment (Fig 2).

**Table 1:** Cephalometric measurements (norm indicates values as per Brazilian norms).

Measurements	Norm	Pre-treatment	Post-treatment
SNA	82°	84°	82°
SNB	80	82°	79°
ANB	2°	2°	1°
SN.GoGn	32°	33°	33°
IMPA	90°	92°	90°
Interincisal angle	131°	126°	130°
Upper lip - S line	0mm	0mm	-1mm
Lower lip - S line	0mm	0mm	-1mm

## TREATMENT OBJECTIVES

The treatment goal for this case was to gain space on maxillary and mandibular arches to promote leveling, alignment and ideal Class I canine and molar relationship. At the same time, it was aimed to promote cautious movements to maxillary right central incisor, which already presented a slight ERR on initial radiographs.

## TREATMENT ALTERNATIVES

Since patient presented moderate crowding on both arches, maxillary and mandibular incisors flaring could be another alternative for this case. However, this option was ruled out for two main reasons. First, lower incisors buccal movement was a periodontal risk considering the initial contour of the roots in the lower incisors (Fig 1), suggesting the lack of bone in this area. Lastly, incisors flaring could jeopardize patient facial profile, which was within normal limits. Thus, it was decided to gain space using RME and/or extracting four premolars.

## TREATMENT PROGRESS

Before starting the new treatment, it was waited four months for spontaneous recovery before bonding fixed appliance (Fig 1). After this period, the patient returned with adequate oral hygiene and the resumed a new orthodontic treatment was resumed. 

**Figure 3:**
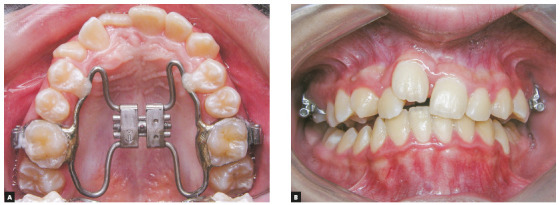
Intraoral photographs before (A) and after (B) RME, showing evidence of the midline diastema after RME.

Due to the maxillary anterior crowding, the primary approach was to create extra space before using orthodontic forces, to align and level the maxillary central incisors. Thus, RME was performed with a Hyrax appliance and a slight midline diastema was obtained (Fig 3). This appliance was used for retention for five months. After this period, as the amount of space gained was insufficient, four premolars were extracted.

**Figure 4:**
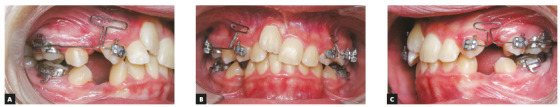
Canine retraction using the segmented arch approach.

**Figure 5:**
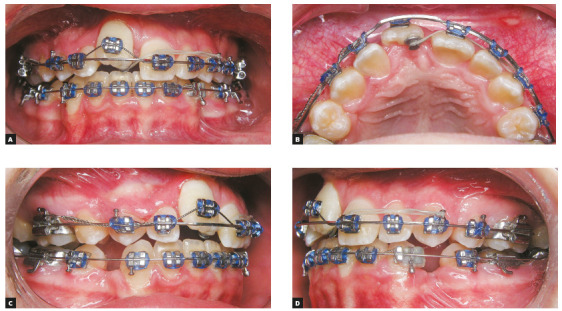
Orthodontic mechanics to correct maxillary right central incisor vertical position and rotation.

**Figure 6:**
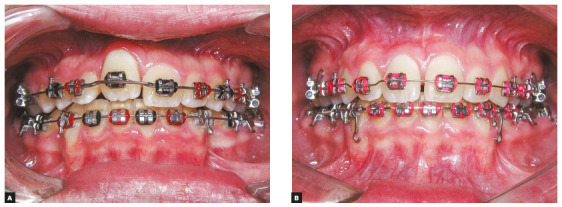
Progress of vertical and axial movement of the maxillary right central incisor after 2 months (A) and 4 months (B) of the incisor bonding.

**Figure 7:**
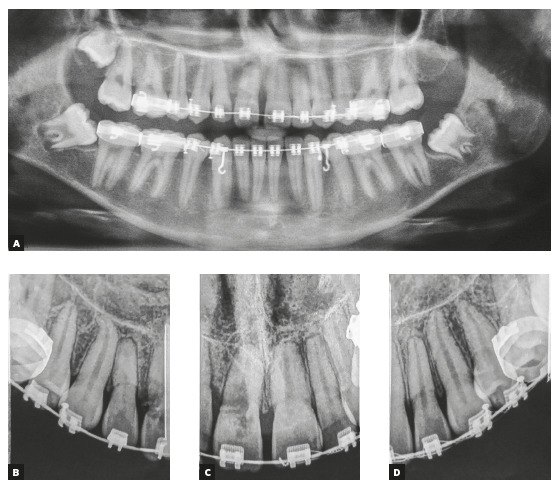
Routine panoramic and periapical radiographs requested after 17 months of treatment. ICR of the maxillary right central incisor was diagnosed.

In order to avoid round-tripping incisor movements, a segmented approach with 0.017*x*0.025-in stainless steel T-loops were used to retract canines (Fig 4). When adequate space was obtained, the remaining brackets were placed, except on the maxillary right central incisor. Sliding mechanics was used to open space for the maxillary right central incisor before using any orthodontic forces on this tooth. After 14 months of treatment, adequate space was obtained to position the maxillary right central incisor, and then a 0.019*x*0.025-in stainless steel (SS) wire was placed to anchor the maxillary teeth. While this approach anchored the maxillary arch, light orthodontic forces with 0.012-in NiTi wire was used to extrude the right central incisor. At the same time, a button was bonded at the palatal side to derotate this tooth (Fig 5). After three months, the heavy wire was kept in place and the 0.012-in NiTi archwire was replaced by a 0.016-in NiTi archwire (Fig 6). Four months later, ideal vertical positioning was accomplished and a 0.018-in SS archwire was placed in the maxillary arch (Fig 6). Routine panoramic and periapical radiographs were requested (Fig 7). 

Radiographic images showed slight apical ERR in the maxillary incisors and a severe asymptomatic ICR in the maxillary right central incisor. Clinically, the tooth exhibited pink discoloration on the cervical region at the palatal surface, thus the patient was referred to endodontic and periodontic specialists for evaluation. The ICR was classified as a Class 4 type, since the lesion had extended beyond the coronal third of the tooth root and tooth extraction was indicated. Since the patient wasn’t old enough to perform the tooth implant, the multidisciplinary team (periodontist, endodontist, prosthodontist and orthodontist) decided to keep the tooth in order to maintain alveolar bone levels, until patient reached a suitable age to perform the extraction and insertion of an osseointegrated implant, avoiding the need for a bone graft surgery. Endodontic treatment was not considered, since literature considers it’s outcome unsatisfactory when applied to Class 4 resorptions.^2^ For this purpose, patient was monitored by the multidisciplinary team since the maxillary right central incisor had the crown structure weakened and would need restorative procedures, if necessary.

## RESULTS

**Figure 8:**
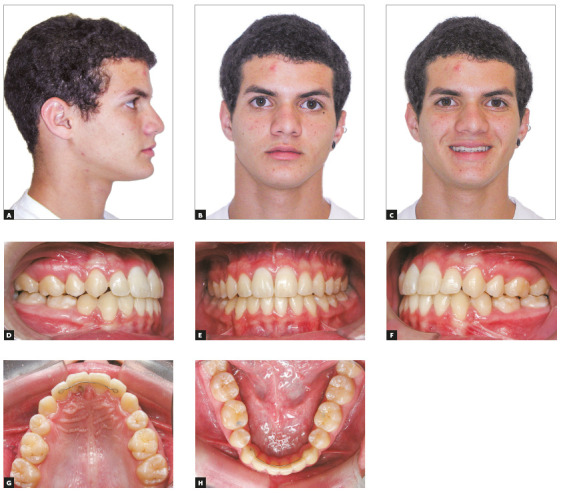
Posttreatment facial and intraoral photographs.

**Figure 9:**
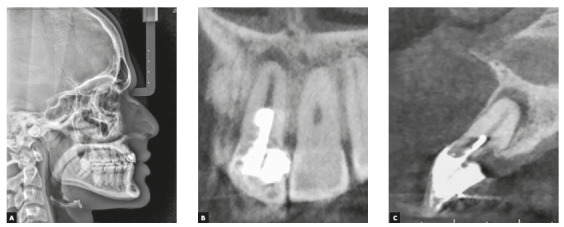
Final lateral radiograph and Cone Beam Computerized Tomograph image of maxillary central incisors on coronal and sagittal views.

The overall treatment duration was 3 years and 9 months. The posttreatment photographs show satisfactory occlusion, Angle Class I molar and canine relationship, coincident dental midlines, ideal overjet and overbite. Bonded maxillary and mandibular 3x3 retainers were installed after treatment (Fig 8). Figure 9 shows final lateral radiograph and Cone Beam Computerized Tomography (CBCT) image, showing the compromised structure of maxillary incisor. Table 1 reveals the final treatment cephalometric measurements.

**Figure 10:**
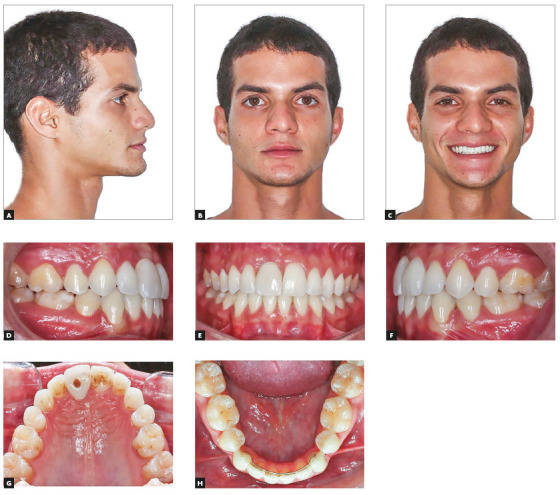
Follow-up intraoral and facial photographs taken 6 years posttreatment and 2 years after implant placement.

**Figure 11:**
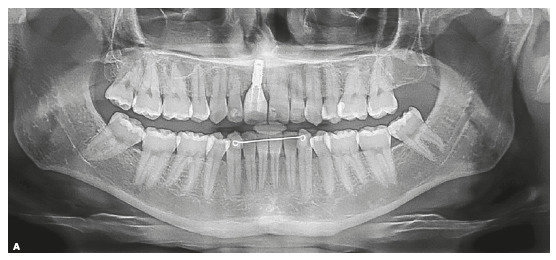
Six years post-treatment panoramic radiograph showing the implant and bone conditions of the maxillary central incisor region.

Six years posttreatment, when the patient was 21 years old, the maxillary right central incisor was extracted and the osseointegrated implant was inserted. At that point, the tooth crown had broke and had been restored with a temporary prosthesis. Figures 10 and 11 show photographs and panoramic radiograph six years after the end of the treatment. Despite of the relapse of mandibular midline shift, there was acceptable stability of the final results.

## DISCUSSION

ICR is an important orthodontic therapy complication. Despite its rare occurrence, the damage to the affected tooth might require removal of the affected tissue, restoration of the defect with filling material, and, in cases in which the tooth structure is severely affected, tooth extraction might be required. The feature that makes this case unique is that the asymptomatic ICR affected a permanent upper central incisor during orthodontic treatment, which was eventually extracted. This unwanted side effect is extremely important to highlight the role of routine radiograph exams during orthodontic treatment.

The affected tooth showed a clinical aspect of trauma, but the patient and his family reported no trauma history. According to the family, the right central incisor had delayed eruption during mixed dentition, when the patient started the previous treatment. Literature has pointed out orthodontic treatment as being the most common risk factor for ICR,^9^ and there is a higher percentage of cases when the two risk factors - orthodontic treatment and occurrence of trauma - were found in combination. However, this event may be underestimated, since patients might not be able to recall trauma to their teeth.^10^

According to Heithersay,^2^ ICR can be classified in four different levels (from Class 1 to 4), according to its extent. The treatment and prognosis of this pathology varies with the degree of the resorptive process. Usually Classes 1, 2 and 3 allow removal of the resorptive tissue and restoration of the defect.^11^ A multidisciplinary approach might be necessary if the resorption involves the root canal system and demands endodontic treatment; or if the lesion is located too far in the apical direction, and requires orthodontic forced eruption to expose the resorption area to allow the filling procedure.^12^ The Class 4 ICR is a large invasive resorption that reaches beyond the coronal third of the root, and its occurrence demands extraction of the tooth.^3^ In the presented case, the tooth exhibited a pink discoloration on the cervical region at the palatal surface, and the periapical radiography revealed coronal radiolucency that extended deeply into the root on apical direction (Fig 8).

This case was conducted with the aim of preventing unnecessary tooth movement, such as round tripping of the incisors, and to avoid unwanted forces during aligning and leveling stages. The maxillary anterior teeth, especially the maxillary right central incisor, were bonded only after adequate space was gained. This approach consisted of RME and subsequent extraction of four premolars. Additionally, light forces were used to extrude the right central incisor. Since controlled mechanics were used and the right central incisor was carefully positioned, the possibility of this orthodontic treatment being associated with the primary etiology of this ICR was not considered. However, it was not possible to make the same statement about the orthodontic mechanics used in the previous treatment. It is unclear whether the previous orthodontic treatment could be an iatrogenic factor and a possible cause of the ICR in this tooth. 

Another important precaution taken during the treatment planning was that after discontinuation of the first treatment, four months were waited for recovery, before resuming the new treatment. This clinic conduct was based on previous studies,^13^ ^-^ ^15^ as several authors have addressed the recovery of dental and periodontal tissues after force application, based on animal research. In addition, Katzhendler and Steigman^16^ highlighted the increased risk of ERR during the retreatment of a previously moved tooth. Although most studies have investigated the apical ERR, this might have played a role in this other type of ERR that affected the cervical area of the tooth - that is, the ICR. 

As the radiographic image of the ICR looks like an Internal Root Resorption (IRR), it’s important to clarify the differential diagnoses of the two pathologies. The ICR is a type of external inflammatory resorption that begins on the cervical area of the tooth; thus, the diagnose is frequently done using radiographs and during clinical examination. The IRR is a pulp disease that causes the resorption of the tooth structure, starting from the root canal. It is also usually asymptomatic and recognized through routine radiographs. Several authors^17^ ^-^ ^19^ have emphasized the difficulty in distinguishing IRR and ICR, especially when the ICR is not accessible by probing and, on radiographic examination, it is projected over the root canal. In order to guide clinicians on radiographic differentiation of the ICR and IRR, Gartner et al.^20^ described IRR as smooth and symmetrically distributed lesions, and ICR as asymmetrical and with borders that are poorly defined. Thus, the clinical examination performed by the endodontic and periodontic specialists was imperative to diagnosis definition.

There is no doubt about the importance of the initial radiographic screening before beginning the orthodontic treatment. For the treatment planning, it is necessary to accurately assess the relationships of the teeth to the jaws, and the jaws to the rest of the facial skeleton. Although the British Orthodontic Society recommendations^21^ for monitoring treatment involves the use of radiographs only to assess unerupted teeth, iatrogenic factors, and at the end of active tooth movement; the use of routine radiographs was essential for the diagnosis of ICR in this case, especially because of its asymptomatic nature. ERR of maxillary incisors is considered an important issue, as demonstrated by the mechanics applied in the current clinical case. However, periapical radiographs of maxillary central incisors could have been taken earlier than 17 months of treatment. Thus, it might have been possible to save this tooth. 

## CONCLUSION

ICR is a possible complication that might occur during orthodontic treatment and few authors have reported such problem. This clinical example highlights two important aspects regarding this issue: the importance of routine follow-up radiographs during treatment, to allow early diagnosis of this asymptomatic problem; and the treatment difficulties related to ICR, such as the need for tooth extraction. 

Since orthodontic treatment has been listed as a possible risk factor to ICR, this article brings important concepts regarding this issue, in order to assist the clinician in dealing with this unwanted situation.
